# Multicenter Clinical Evaluation of ETEST Plazomicin (PLZ) for Susceptibility Testing of Enterobacterales

**DOI:** 10.1128/JCM.01831-21

**Published:** 2022-01-19

**Authors:** Laurine S. Blanchard, Alex Van Belkum, Dominique Dechaume, Thomas P. Armstrong, Christopher L. Emery, Yun X. Ying, Michael Kresken, Marion Pompilio, Diane Halimi, Gilles Zambardi

**Affiliations:** a bioMérieux SA, Marcy l’Etoile, France; b bioMérieux, SA., La Balme-les-Grottes, France; c bioMérieux Inc., Hazelwood, Missouri, USA; d Indiana University School of Medicine, Indianapolis, Indiana, USA; e Quest Diagnostics, Lewisville, Texas, USA; f Antiinfectives Intelligence GmbH, Cologne, Germany; Medical College of Wisconsin

**Keywords:** antimicrobial susceptibility testing, gradient methods, Plazomicin, ETEST, Enterobacterales, plazomicin resistance mechanisms

## Abstract

Plazomicin (PLZ), brand name ZEMDRI (Cipla Therapeutics), is a novel aminoglycoside antibiotic approved by the U.S. Food and Drug Administration (FDA) for treatment of complicated urinary tract infections including pyelonephritis. ETEST® is a gradient diffusion method that represents an alternative to the more laborious broth micro-dilution (BMD) method for performing antimicrobial susceptibility testing (AST). A multi-center evaluation of the performance of the new ETEST PLZ (bioMérieux) was conducted in comparison with BMD following FDA and International Standards Organization (ISO) recommendations using FDA-defined breakpoints. Clinical isolates of Enterobacterales (*n* = 598) were included. Fifty-three isolates were resistant to PLZ according to BMD. Overall, the ETEST PLZ demonstrated 99.0% essential agreement (EA), 92.8% category agreement (CA), 1.9% very major errors (VME), 0% major errors (ME), and 7.0% minor errors (mE) with both clinical and challenge isolates of Enterobacterales. The VME was found for a single Serratia marcescens strain. Individual species demonstrated EA rates ≥ 90%. In conclusion, we report that ETEST PLZ represents an accurate tool for performing PLZ AST of Enterobacterales.

## INTRODUCTION

Plazomicin (PLZ) is a novel aminoglycoside approved by the U.S. Food and Drug Administration (FDA) for treatment of complicated urinary tract infections (cUTI, including acute pyelonephritis) in adults due to multidrug-resistant carbapenem-resistant Enterobacterales (CRE) or extended-spectrum beta-lactamase (ESBL) producing bacteria (1–3). Despite the FDA Black Box Warning for aminoglycoside class effects, PLZ exhibited a favorable safety profile with decreased adverse effects in the largest in-human trials (4, 5). *In vitro* activity of PLZ in comparison with that of other aminoglycosides was found to be excellent with 92.3% to 98.0% overall susceptibility in Enterobacterales including CRE (6–9). Recently, PLZ was also shown to be active against certain multidrug-resistant (MDR) and extensively drug-resistant (XDR) Gram negative pathogens for which limited treatment options are left (10, 11). In the Combating Antibiotic-Resistant Enterobacteriaceae (CARE) trial, serious adverse events (including death) occurred less frequently in the PLZ group than in the colistin group of patients with CRE infections (5).

PLZ maintains activity against Enterobacterales that express resistance mechanisms to other antibiotic classes, including metallo-β-lactamases (MBL) (12). However, the presence of a 16S rRNA methyltransferase (16S-RMTase) in CRE abrogates all clinical use of aminoglycosides including PLZ (12, 13). Certain environmental species of aminoglycoside-producing bacteria may also produce a 16S rRNA methyltransferase (16S-RMTase) which post-transcriptionally methylates residue G1405 of 16S rRNA resulting in high-level intrinsic resistance to gentamicin, tobramycin, amikacin, and plazomicin. Strains that produce 16S-RMTase are frequently MDR or even XDR. Ongoing spread of this mechanism may further limit options for adequate treatment of infections (14). A significant proportion of *bla*_NDM_ and *bla*_OXA48_-like-expressing strains also harbor aminoglycoside-modifying enzymes implied in PLZ resistance. PLZ has shown some activity against MBL-CRE pathogens. However, *bla*_NDM_-expressing isolates often co-express resistance to PLZ due to aminoglycoside modifying enzymes AAC(2’)-Ia and APH(2”)-IVa limited in their distribution to Providencia stuartii and Enterococci, respectively (15). Additionally, an integron-borne gene cassette encoding a protein that conveys high-level resistance against aminoglycosides has been identified recently. This *gar* gene, although not common in clinical isolates, may ultimately reduce the usefulness of PLZ (16). Consequently, continuous antimicrobial susceptibility testing (AST) is appropriate in settings where the prescription of PLZ is anticipated. Our objective was to conduct a multi-center evaluation of the analytical performance of ETEST® PLZ (bioMérieux, Marcy-l’Étoile, France) compared with reference broth micro-dilution (BMD) testing. ETEST PLZ was recently cleared for *in vitro* diagnostic (IVD) use by the U.S. FDA (17). It can be used to determine the MIC of Plazomicin against the following microorganisms: Escherichia coli, Klebsiella pneumoniae, Proteus mirabilis, Enterobacter cloacae, Citrobacter freundii, Citrobacter koseri, Klebsiella aerogenes, Klebsiella oxytoca, Morganella morganii, Proteus vulgaris, Providencia stuartii, and Serratia marcescens.

## MATERIALS AND METHODS

### Participating institutes and ethical clearance.

The study was conducted at four different sites, including two in the United States at the Indiana University School of Medicine (IUSM, Indianapolis, IN, USA) and Quest Diagnostics (Quest, Lewisville, TX, USA) and two in Europe at Anti-infectives Intelligence, GmbH (AI, Cologne, Germany) and bioMérieux SA (Marcy l’Etoile, France). Each American study site performing testing on clinical strains acquired local institutional review board approval or waiver thereof prior to study initiation.

### Clinical bacterial isolates.

In the clinical study, 518 clinical isolates were tested among the four sites (170 at AI, 170 at Quest, 170 at IUSM, and eight at Marcy). The overall distribution of these clinical isolates by source of infection was as follows: urine, 45.0%; wounds, 12.6%; respiratory tract, 9.7%; blood, 9.5%; body fluid, 3.1%; digestive tract, 2.7%; and others (sample types from which ≤ 9 isolates each were obtained), 8.7%. The source of infection was unknown for another 8.9% of the clinical isolates because the strains were collected from stock collection or obtained from the Centers for Disease Control and Prevention (CDC, Atlanta, USA). Clinical isolates were acquired from routine cultures processed in the clinical laboratory at each of the trial sites. Clinical isolates were identified to the genus and species level using a MALDI-TOF technique. The technician performing the clinical trial testing did not have prior knowledge of any contemporary clinical isolate’s susceptibility results. Duplicate isolates from the same patient were excluded from the clinical trial. Of the 518 clinical isolates, 286 (55.2%) were contemporary isolates (tested within 6 months from isolation in culture, not preselected, and if frozen, minimally subcultured) and 232 (44.8%) were stock (frozen with no time constraints and minimally subcultured). Among clinical isolates, 35 (6.8%) were resistant to PLZ by BMD according to FDA interpretive criteria (≤ 2, susceptible; 4 intermediate; ≥ 8, resistant) including eight from the CDC panel.

### Characterization of PLZ challenge set isolates.

Among 80 challenge isolates (clinical isolates of Enterobacterales characterized using WGS) tested, 18 were resistant to PLZ by BMD according to FDA breakpoints. The characterization of the challenge isolates is listed in Table S1. Genes related to β-lactamases and aminoglycoside resistance (RNA 16S methylase and aminoglycoside modifying enzymes) have been identified. Other mechanisms such as efflux have not been examined in this study. The presence of the *gar* gene was not determined in this study because it was unknown when this research was performed. Ten of the 18 isolates harbored mutant RNA methylases (4 *armA*, 4 *rmtB*, 1 *rmtC*, and 1 *rmtF*).

### Study setting and design.

The performance of the ETEST PLZ was compared with that of the BMD reference method following Clinical and Laboratory Standards Institute (CLSI) M07-Ed11 (18) and International Standards Organization (ISO) ISO 20776-1 (19) standards. The study design included four performance components: (i) a challenge study, (ii) a clinical study, (iii) a quality control (QC) study, and (iv) a reproducibility study. These four substudies included Enterobacterales isolates of the following species: Citrobacter koseri, Citrobacter freundii, Enterobacter cloacae, Escherichia coli, Klebsiella aerogenes, Klebsiella oxytoca, Klebsiella pneumoniae, Morganella morganii, Proteus mirabilis, Proteus vulgaris, Providencia stuartii, and Serratia marcescens. Purity of inoculum was checked for all isolates tested, regardless of the study component. Inoculum density was verified by colony count for all quality control replicates, all reproducibility tests, and 10% of the contemporary clinical isolates following FDA guidance (20, 21). Challenge, clinical, reproducibility, and quality control studies took place in Marcy, while clinical, reproducibility, and quality control studies took place at IUSM and Quest. AI performed clinical and quality control studies. Resistance phenotypes of the challenge set isolates to aminoglycosides and beta-lactams were determined using Vitek 2. Challenge set isolates were also characterized using whole genome sequencing (WGS).

### Susceptibility testing methodology.

**FIG 1:**
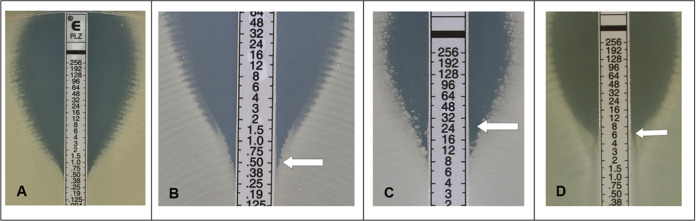
Examples of isolate testing with ETEST PLZ. Examples of organisms tested and their MIC readings are shown in Panels A to D. (A) E. coli ATCC 25922™, MIC = 0.5 μg/Ml. (B) Reading at the bottom of the “dip” if colonies are absent. MIC = 0.5 μg/mL. (C) Reading at complete inhibition of growth, including haze and microcolonies. MIC = 24 μg/mL. (D) For Proteus spp., read at complete inhibition of growth, even if swarming is observed. MIC = 6 μg/mL.

A visual calibrator was used to prepare a 0.5 McFarland inoculum (for nonmucoid isolates) in 0.85% sterile saline from 18 h to 24 h colony growth on tryptic soy or Columbia agar plates supplemented with 5% sheep blood. For mucoid isolates, a 1.0 McFarland standard inoculum was prepared for ETEST and a 0.5 McFarland suspension was prepared for BMD. Within 15 min of preparation, a sterile cotton swab moistened with the standardized bacterial suspension was inoculated manually or automatically using the Retro C80™ rota-plater on BBL™ Mueller-Hinton II agar plates (BD; Sparks, MD) and ETEST strips were applied to plates with an applicator (Nema C88™ vacuum pen, bioMérieux; Durham, NC) or forceps. Plates were incubated in ambient air at 35 ± 2°C and read after 16 h to 20 h of incubation. The MIC was read at the concentration of PLZ showing complete inhibition of growth (bactericidal reading) as described by the ETEST PLZ instructions for use (22). An example of MIC determination is shown in Fig. 1. An MIC falling between two dilutions was rounded up to the next highest value. Nondoubling MIC values (e.g., 0.75, 3) were rounded up if necessary to the standard doubling dilution before categorization. Hazy growth as well as the presence of macro- or micro-colonies within 3 mm from the strip were read as growth as well. BMD was performed in frozen 96-well plates prepared at the bioMérieux facilities (La Balme les Grottes, France) in compliance with the directions in CLSI M07-Ed11 (18) and ISO 20776-1 (19) standards. The BMD panels consisted of 2-fold dilutions of PLZ in cation-adjusted Mueller-Hinton broth. Prepared panels were concentrated twice to reach a final concentration after inoculation ranging from 0.016 to 256 μg/mL. Each batch produced was controlled by inoculating several panels selected at the beginning, at the middle, and at the end of production with the QC strains recommended by the CLSI M100 standard (23). The panels were then frozen at −80°C and shipped in aluminum pouches on dry ice and constant monitoring of the temperature during transportation to all clinical trial sites. Prior to use, BMD panels were completely thawed at room temperature for 30 min to 1 h. Using a repeating pipette, BMD panels were inoculated with 50 μL per well of a 100-fold dilution of the original bacterial suspension in BBL™ Mueller-Hinton II broth, cation-adjusted (BD; Sparks, MD) of the same 0.5 McFarland suspension used for ETEST PLZ and incubated at 35 ± 2°C in ambient air for 16 h to 20 h. An aliquot was removed from each growth control well of the BMD panels, inoculated on blood agar and assessed for purity after 20 h to 24 h and 44 h to 48 h of incubation. Inoculum density checks were performed by plating 100 μL of a 1:1,000 dilution of the growth control from BMD panels onto a blood agar plate which was subsequently incubated at 35 ± 2°C in ambient air for 18 h to 48 h. After incubation, colony counts were recorded and used to calculate inoculum density.

### Reproducibility study.

Ten on-scale isolates provided by bioMérieux (C. freundii, *n* = 1; K. aerogenes, *n* = 1; E. cloacae, *n* = 1; E. coli, *n* = 1; K. pneumoniae, *n* = 3; P. mirabilis, *n* = 1; P. stuartii, *n* = 1; S. marcescens, *n* = 1) were tested at the IUSM, Quest, and Marcy CA study sites. Isolates were subcultured twice on blood agar before testing. Each isolate was tested in triplicate on three different days. Three separate 0.5 McFarland suspensions of each isolate were prepared in normal saline for the ETEST and inoculum density check. Different lots of Mueller-Hinton plates from the same manufacturer (BD) were used. The results from all three sites were used to compute a modal MIC value for each strain used in the reproducibility study (if there was no modal value, the median was used). The total number of results within one doubling dilution of the mode or median was used to calculate the reproducibility rate as the percentage of total number of tests. Best case calculations for reproducibility assumed off-scale values were within one doubling dilution of the mode. Worst case reproducibility assumed off-scale values were not within one doubling dilution.

### Quality control study.

QC testing was performed at each study site every day of comparative testing and a minimum of 20 times at each site with the following organisms: Escherichia coli ATCC 25922™, Enterococcus faecalis ATCC 29212™, Pseudomonas aeruginosa ATCC 27853™, and Staphylococcus aureus ATCC 29213™. QC strains were subcultured twice on blood agar before testing. QC ranges described by CLSI M100 Ed-29 were verified on each day of clinical or challenge testing and an inoculum density check was conducted for all QC tests according to CLSI M07-Ed11 guideline (18, 23). Results were considered invalid if QC results were out of range. QC performance for ETEST PLZ was calculated as the percentage of results within the expected range.

### Clinical and challenge studies.

Five-hundred and 18 clinical isolates of Enterobacterales and 80 challenge isolates were evaluated for PLZ susceptibility using ETEST PLZ and reference BMD simultaneously using the methods described above. Clinical isolates were tested at the four sites while challenge isolates testing was performed entirely at Marcy. Every isolate was subcultured twice on blood agar before testing. No duplicate isolates from the same patient were included. A single 0.5 McFarland suspension was prepared for ETEST and BMD testing. For mucoid isolates, a 1.0 McFarland suspension was prepared for ETEST and the 0.5 McFarland was used for BMD. Inoculum density check as described above was performed on a minimum of 10% of contemporary clinical isolates at each study site.

### Data analysis.

Clinical and challenge data were combined in the performance evaluation. MIC values were interpreted using FDA breakpoints (24). Performance was evaluated using U.S. FDA performance criteria of EA and CA ≥ 90%, MRE ≤ 3.0%,VME rate ≤ 2.0% and < 30% trend (20, 21). EA was defined as the percentage of total isolates where the test and reference methods were within one doubling-dilution of each other; CA was defined as the percentage of total test results in agreement within category (susceptible, intermediate and resistant) of the reference method using FDA interpretative criteria as indicated; the VME rate was defined as the percentage of isolates interpreted as resistant by the reference method which were susceptible by the ETEST method. The ME rate was defined as the percentage of isolates interpreted as susceptible by the reference method which were resistant by the ETEST method. The mE rate was defined as the percentage of total isolates where the reference method interpretation was resistant or susceptible and the ETEST interpretation was intermediate or vice versa. ETEST MIC values from challenge and clinical results in the frequency table were separated in three categories, (i) clearly at least one dilution lower than the reference method MIC value; (ii) Equal to the reference method MIC values, and (iii) Clearly at least one dilution higher than the reference method MIC values. Trending was defined as an upward or downward change associated with increased resistance or increased susceptibility. Trending may be used to compare results between different susceptibility testing methods to assess bias that would not be evident using EA or CA, unless larger numbers of organisms were evaluated. A trend ≥ 30% must be reflected in the labeling.

## RESULTS

### Quality control of ETEST PLZ.

To confirm the QC range of the ETEST PLZ, two QC organisms were tested a minimum of 20 times with ETEST at each site and four were tested a minimum of 20 times with the BMD method throughout the study at all study sites as described in the methods. One-hundred percent of BMD and ETEST results for E. coli ATCC 25922™ and E. faecalis ATCC 29212™ were within the expected range defined by the CLSI M100 (23) standard; hence, meeting both FDA and ISO requirements. BMD results for S. aureus ATCC 29213™ were out of range once resulting in 98.78% (81/82) of all QC results being within the required range. All reference BMD QC results for P. aeruginosa ATCC 27853™ were within range (81/81, 100%).

### Reproducibility of ETEST PLZ.

Evaluation in triplicate for 3 days resulted in 90 determinations per site, 270 measurements in total. Mode values of PLZ MICs for each isolate tested across all sites are shown in Table 1. The reproducibility rate of all strains between sites (270/270) and within site (90/90) was 100% within ± 1 doubling dilution of strain-specific modal values.

**TABLE 1:** Reproducibility of ETEST PLZ

Organism	MIC test mode (μg/mL)	Doubling dilution from the mode						
		Off-scale	−2	−1	0	+1	+2	Off-scale
Citrobacter freundii	8			12	15			
Enterobacter cloacae	0.25				27			
Escherichia coli	2				27			
Klebsiella aerogenes	0.5				24	3		
Klebsiella pneumoniae	0.25				25	2		
Klebsiella pneumoniae	4			6	21			
Klebsiella pneumoniae	8				21	6		
Proteus mirabilis	16			7	20			
Providencia stuartii	2			3	24			
Serratia marcescens	4				26	1		
Total		0	0	28	230	12	0	0

### Challenge and clinical performance.

A total of 80 isolates from the Enterobacterales family were included in the challenge study and tested at the Marcy study site (the composition of the challenge set appears in Table 2 and Table S1). Eighteen (22.5%) Enterobacterales isolates were resistant to PLZ by BMD according to FDA breakpoints. Among all isolates, ETEST PLZ demonstrated 100.0% (80/80) EA and 90.0% (72/80) CA. No VME and ME were detected, but 10.0% (8/80) mE were observed (Table 2). EA for clinical isolates was 98.8% (512/518), CA was 93.2% (483/518) with 2.9% (1/35) VME, 0% (0/423) ME, and 6.6% (34/518) mE rates.

**TABLE 2:** Clinical and challenge performance of ETEST PLZ^*a*^

	Organism	Total	EA	%EA	Total evaluable	EA of evaluable	CA	%CA	#R	#VME	#ME	#min
Challenge	C. freundii	6	6	100.0%	6	6	6	100.0%	0	.	0	0
	*C. koseri*	3	3	100.0%	3	3	3	100.0%	0	.	0	0
	E. cloacae	10	10	100.0%	10	10	10	100.0%	0	.	0	0
	E. coli	13	13	100.0%	8	8	10	76.9%	5	0	0	3
	K. aerogenes	6	6	100.0%	6	6	6	100.0%	1	0	0	0
	K. oxytoca	7	7	100.0%	7	7	6	85.7%	0	.	0	1
	K. pneumoniae	17	17	100.0%	13	13	16	94.1%	7	0	0	1
	M. morganii	3	3	100.0%	3	3	2	66.7%	2	0	0	1
	P. mirabilis	4	4	100.0%	4	4	4	100.0%	2	0	0	0
	P. vulgaris	5	5	100.0%	5	5	3	60.0%	0	.	0	2
	P. stuartii	2	2	100.0%	1	1	2	100.0%	1	0	0	0
	S. marcescens	4	4	100.0%	4	4	4	100.0%	0	.	0	0
	Subtotal	80	80	100.0%	70	70	72	90.0%	18	0	0	8
Clinical	C. freundii	53	53	100.0%	52	52	53	100.0%	1	0	0	0
	*C. koseri*	31	30	96.8%	31	30	31	100.0%	0	.	0	0
	E. cloacae	50	50	100.0%	48	48	50	100.0%	2	0	0	0
	E. coli	65	63	96.9%	64	62	65	100.0%	1	0	0	0
	K. aerogenes	32	32	100.0%	31	31	32	100.0%	1	0	0	0
	K. oxytoca	32	32	100.0%	31	31	31	96.9%	1	0	0	1
	K. pneumoniae	72	72	100.0%	63	63	72	100.0%	11	0	0	0
	M. morganii	28	27	96.4%	28	27	19	67.9%	3	0	0	9
	P. mirabilis	59	59	100.0%	57	57	50	84.7%	6	0	0	9
	P. vulgaris	29	29	100.0%	29	29	26	89.7%	1	0	0	3
	P. stuartii	33	33	100.0%	30	30	24	72.7%	7	0	0	9
	S. marcescens	34	32	94.1%	34	32	30	88.2%	1	1	0	3
	Subtotal	518	512	98.8%	498	492	483	93.2%	35	1	0	34
Combined	Total	598	592	99.0%	568	562	555	92.8%	53	1	0	42

a EA, essential agreement; CA, category agreement; R, resistant; VME, very major error; ME, major error; min, minor error.

**FIG 2:**
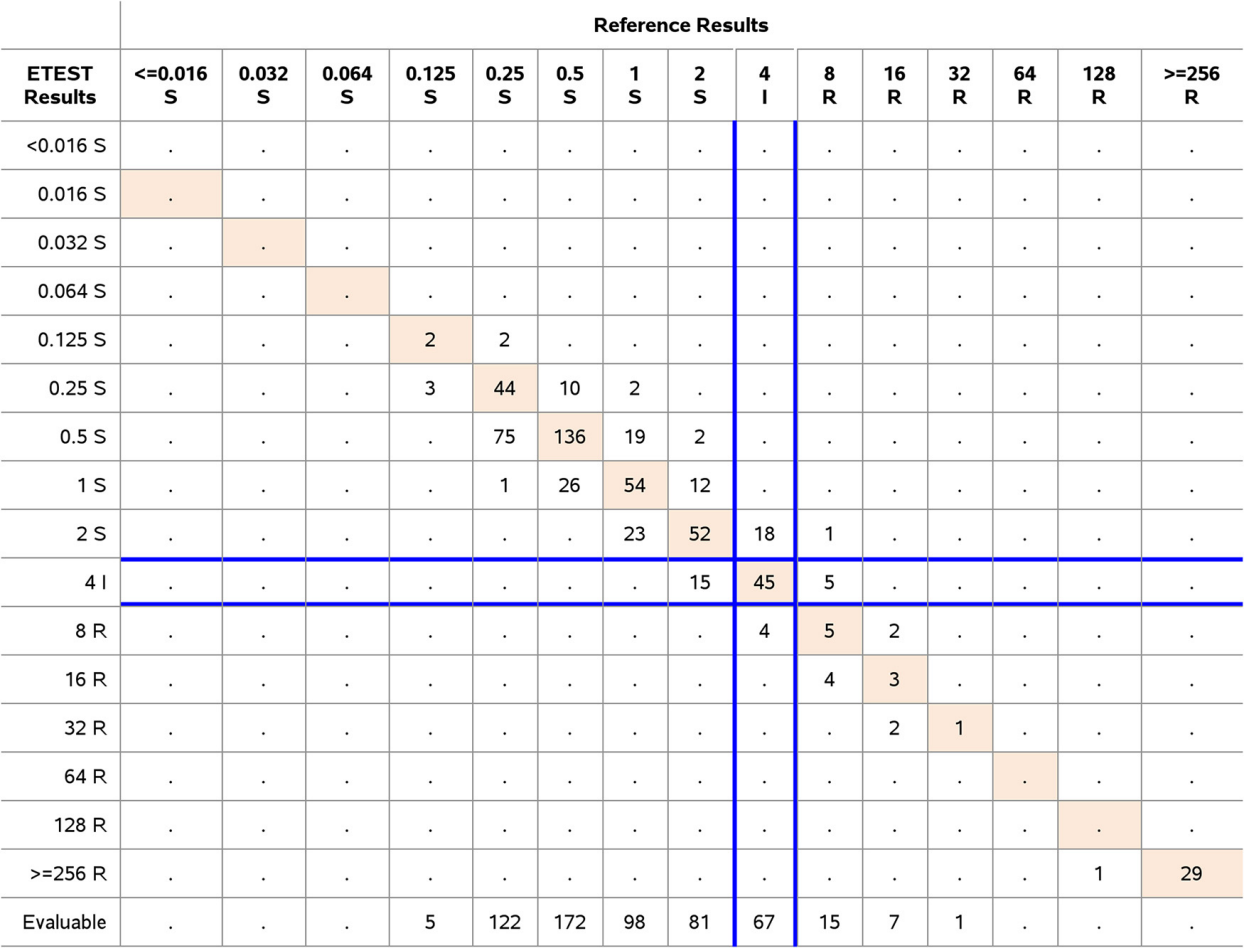
Frequency table for Enterobacterales. Clinical and challenge isolates of Enterobacterales (*n* = 598) were tested for PLZ susceptibility testing with ETEST PLZ and the BMD reference method. The number of isolates with exact MIC agreement between both ETEST and BMD reference method are shown in orange. FDA categories for Enterobacterales and PLZ are written in the header.

Evaluation of the performance of the ETEST PLZ included data for 598 isolates from the challenge and clinical studies were combined. The distribution of species, resistance to PLZ, and performance determined using FDA breakpoints are shown in Fig. 2 and Table 2. The rate of clinical isolate resistance to PLZ determined by ETEST for Enterobacterales was 9.8% according to FDA breakpoints. Overall performance of ETEST PLZ with all clinical and challenge isolates of all species from the Enterobacterales family was 99.0% EA, 92.8% CA, 1.9% VME, and 0.0% ME. The majority of individual species demonstrated CA > 90% with the exception of members of the *Morganellaceae* family including M. morganii (67.7% CA), P. mirabilis (85.7% CA), P. vulgaris (85.3% CA), and P. stuartii (74.3% CA). S. marcescens also showed a CA < 90% (89.5%). The performance is acceptable because the EA was > 90% and all categorical errors were minor and within essential agreement. One VME was observed, a S. marcescens. The ETEST PLZ MIC was 2 μg/mL, the BMD MIC was 8 μg/mL. This isolate was not evaluated for any resistance mechanism. Upon repeat testing in triplicate, the results were as follows, ETEST PLZ 1, 4, and 2; and BMD 4, 4, and 4. Repeat test results were within CA (one third) or represented mE (two thirds). The FDA does not allow categorical error resolution. The initial results were retained for the FDA analysis. This VME was associated with the only resistant isolate for this species as determined by the reference BMD during the initial testing. Consequently, the VME rate was 100% for S. marcescens. Therefore, a limitation was added concerning the absence of resistant isolates for this species in addition to the one for *C. koseri*. Nevertheless, the ETEST PLZ met the criterion for VMEs for the Enterobacterales (1.9%, 1/53).

Trend analyses were calculated for overall species and for each species following FDA guidance (20, 21). A trend to overestimate ETEST PLZ MIC values was observed for K. aerogenes and K. pneumoniae compared with the CLSI reference BMD method, respectively, 35.14%; 95% CI (15.48% to 51.84%) and 44.16%; 95% CI (31.00% to 55.53%). This trend did not have any impact on the CA and EA for these species so no footnote will be proposed. A trend to underestimate ETEST PLZ MIC values was observed for M. morganii compared with the CLSI reference BMD method: −35.48%; 95% CI (–53.40% to –14.40%). This trend is noted in a footnote of the postclinical trial version of the U.S. ETEST PLZ Instructions for use (section Performance Characteristics). No trend was observed for overall species and species other than K. aerogenes, K. pneumoniae and M. morganii.

## DISCUSSION

PLZ acts by binding to bacterial 30S ribosomal subunit, thereby inhibiting protein synthesis. PLZ has concentration-dependent bactericidal activity as measured by time-kill studies. *In vitro* studies demonstrated a plazomicin postantibiotic effect ranging from 0.2 h to 2.6 h at 2× MIC against *Enterobacteriaceae* (25). More precise tools for defining aminoglycoside MICs for such strains are still needed. Here, we evaluated the performance of ETEST PLZ compared with the BMD reference method according to FDA breakpoints for Enterobacterales isolates. Overall, ETEST PLZ exceeded FDA performance criteria demonstrating 99.0% EA and 92.8% CA with only a single, non-reproducible VME and no ME observed for clinical and challenge Enterobacterales. The clinical performance of the ETEST PLZ test was found useful for determining the MIC of PLZ for Enterobacterales, including those that produce extended spectrum beta-lactamase (ESBL) or high levels of AmpC β-lactamases; carbapenem-resistant Enterobacterales (CRE, including those that produce metallo-β-lactamases, K. pneumoniae carbapenemase (KPC), or oxacillinase-48 (OXA-48); and aminoglycoside-resistant strains. Among the 518 clinical isolates tested including a part of the population selected for its resistance, the overall susceptibility to PLZ according to the reference method was 81.7%. The mode MIC was low, at 0.5 μg/mL (susceptible). PLZ, as an aminoglycoside antibiotic, provides a treatment option for cUTI with multidrug-resistant Enterobacterales.

PLZ resistance is rare among Enterobacterales worldwide but it is known in metallo-β-lactamase-producing Enterobacterales and more generally on CRE in the presence of a 16S-RMTase (12). Resistance to aminoglycosides includes production of aminoglycoside modifying enzymes (AMEs), alteration of the ribosomal target through production of 16S rRNA methyltransferases, upregulation of efflux pumps, and reduced permeability into bacterial cell due to loss of outer membrane porins. PLZ is not inhibited by most AMEs known to affect gentamicin, amikacin, and tobramycin, including acetyltransferases (AACs), phosphotransferases (APHs), and nucleotidyltransferases (ANTs). PLZ may have reduced activity against Enterobacterales that overexpress certain efflux pumps (e.g., acrAB-tolC) or lower expression of porins (e.g., ompF or ompK36). PLZ has no *in vitro* activity against streptococci (including Streptococcus pneumoniae), enterococci (including Enterococcus faecalis, E. faecium), anaerobes, Stenotrophomonas maltophilia, and Acinetobacter spp. and it has variable activity against Pseudomonas aeruginosa (26). Activity of PLZ was demonstrated *in vitro* against *Enterobacteriaceae* in the presence of certain beta-lactamases, including extended-spectrum beta-lactamases (TEM, SHV, CTX-M, AmpC), serine carbapenemases (KPC-2, KPC-3), and oxacillinase (OXA-48). Bacteria producing metallo-betalactamases often co-express 16S rRNA methyltransferase, conferring resistance to PLZ (25). In keeping with this epidemiology, a limitation of our present study is the lack of *C. koseri* and S. marcescens isolates resistant to PLZ. (27)

Strengths of the current study are that it includes the use of a collection of isolates with different mechanisms of resistance to different β-lactam and aminoglycoside antibiotics (see Table S1), the large number of clinical isolates (598) included, the multicenter evaluation involving three clinical trial sites in independent geographically diverse study sites and the use of a CLSI BMD reference method with a standardized and validated preparation of panels. Limitations of the current study are the lack of resistant *C. koseri* and S. marcescens isolates, the low CA for *Morganellaceae* and S. marcescens, the trend to overestimate MICs when testing ETEST PLZ with K. aerogenes and K. pneumoniae and to underestimate MICs when testing Morganella morganii. The results of this trial supported FDA clearance of ETEST PLZ. This is the first study comparing the performance for this new ETEST PLZ strip to that of the standard BMD in a clinical setting. Overall, we report that the ETEST PLZ demonstrated acceptable performance for Enterobacterales compared with the reference BMD method. Resistance to PLZ in Enterobacterales does occur, highlighting the need for susceptibility testing when this antimicrobial agent has to be used clinically. Given the relative ease of use and the strong performance characteristics, our data support the use of bioMérieux ETEST PLZ in routine clinical practice.
