# Supplementary material for: Multicenter Clinical Evaluation of ETEST Plazomicin (PLZ) for Susceptibility Testing of Enterobacterales
Source: J Clin Microbiol. 2022 Jan 19;60(1):e01831-21. doi: 10.1128/JCM.01831-21 (PMC8769719; doi:10.1128/JCM.01831-21)
Supplement: Supplemental file 1 — Table S1. Download JCM.01831-21-s0001.pdf, PDF file, 0.7 MB [file jcm.01831-21-s0001.pdf]

Table S1. PLZ Challenge Set Characterized Strains Listing

Strain	Organism	Genotype beta-lactams (*genes* identified by WGS)	Genotype aminoglycosides (PROTEINS and *genes* identified by WGS)	Beta-lactam and aminoglycoside [GEN, TOB, NET, AMI only])	BMD PLZ MIC (µg/mL)
1	*Citrobacter koseri*	*bla*$_{TEM-110}$<br>*bla* $_{MAL-CKO\ Like}$	AAC(3)-IIa<br>APH(3'')-Ib<br>APH(6)-Id<br>ANT(3'')-Ia	Acquired Pase<br>Aminoglycoside resistant (AME*)	2
2	*Citrobacter koseri*	*bla*$_{TEM-3}$<br>*bla* $_{MAL-CKO\ Like}$	AAC(6')-Ib like<br>ANT(3'')-Ia	ESBL<br>Aminoglycoside resistant (AME)	0.25
3	*Klebsiella aerogenes*	*bla*$_{TEM-1}$	AAC(3)-IIa<br>AAC(6')-Ib like<br>ANT(3'')-Ia<br>APH(3'')-Ib<br>APH(6)-Id	Carbapenem Resistant (impermeability)<br>Aminoglycoside Resistant (AME)	0.5
4	*Enterobacter cloacae*	*bla* $_{MIR}$	None	Carbapenem Resistant (impermeability)<br>Aminoglycoside wild	0.25
5	*Escherichia coli*	*bla*$_{TEM-2}$	APH(3')-Ib	Acquired Pase<br>Aminoglycoside wild	0.25
6	*Klebsiella pneumoniae*	*bla*$_{TEM-11}$<br>*bla* $_{SHV-83}$	AAC(3)-IIa<br>ANT(3'')-Ia	ESBL and/or SHV hyperproduction<br>Aminoglycoside Resistant (AME)	0.25
7	*Escherichia coli*	*bla*$_{TEM-21}$	AAC(3)-IIa<br>AAC(6')-Ib like	ESBL<br>Aminoglycoside Resistant (AME)	1
8	*Escherichia coli*	*bla*$_{TEM-29}$<br>*bla*$_{CTX-M}$	AAC(3)-IIa<br>ANT(3'')-Ia<br>APH(3'')-Ib<br>APH(6)-Id	ESBL<br>Aminoglycoside Resistant (AME)	0.5
9	*Klebsiella aerogenes*	*bla*$_{TEM-24}$<br>*bla*$_{AMPC}$	AAC(6')-Ib like<br>ANT(3'')-Ia	ESBL<br>Aminoglycoside Resistant (AME)	1
10	*Citrobacter freundii*	*bla* $_{CMY-2\ like}$<br>*bla*$_{TEM-3}$<br>*bla*$_{OXA-10\ like}$	AAC(3)-IIa<br>AAC(6')-Ib like<br>AAC(6')-If<br>ANT(3'')-Ia	ESBL<br>Aminoglycoside Resistant (AME)	0.5
11	*Klebsiella pneumoniae*	*bla*$_{TEM-1}$<br>*bla*$_{SHV}$ *(multiple alleles including one for an ESBL)*<br>*bla*$_{KPC}$<br>*bla*$_{OXA-9\ Like}$<br>*bla*$_{FOX}$<br>*bla*$_{CARB-1\ like}$	AAC(3)-IVa<br>AAC(6')-Ib like<br>ANT(2'')-Ia<br>ANT(3'')-Ia<br>APH(3')-Ia<br>APH(4)-Ia	ESBL + Acquired Case + Carbapenemase (KPC)<br>Aminoglycoside Resistant (AME)	0.25
12	*Citrobacter freundii*	*bla*$_{CMY-2}$ *like*	None	High level Case<br>Aminoglycoside wild	1

Strain	Organism	Genotype beta-lactams (*genes* identified by WGS)	Genotype aminoglycosides (PROTEINS and *genes* identified by WGS)	Beta-lactam and aminoglycoside [GEN, TOB, NET, AMI only])	BMD PLZ MIC (µg/mL)
13	*Escherichia coli*	*bla*$_{\text{TEM-1}}$<br>*bla*$_{\text{KPC}}$<br>*bla*$_{\text{OXA-1 like}}$	AAC(6')-IIc<br>ANT(3'')-Ia<br>APH(3'')-Ib<br>APH(6)-Id	Carbapenemase (KPC)<br>Aminoglycoside Resistant (AME)	2
14	*Enterobacter cloacae*	*bla*$_{\text{VIM}}$	AAC(6')-II<br>ANT(3'')-Ia<br>APH(3')-Ia<br>APH(3'')-Ib<br>APH(6)-Id	Carbapenemase (MBL)<br>Aminoglycoside Resistant (AME)	0.5
15	*Enterobacter cloacae*	*bla*$_{\text{IMP}}$<br>*bla*$_{\text{ACT}}$	AAC(6')-Ib like	Carbapenemase (MBL)<br>Aminoglycoside Resistant (AME)	0.25
16	*Serratia marcescens*	*bla*$_{\text{VIM}}$<br>*bla*$_{\text{SEC}}$	AAC(6')-Ic<br>AAC(6')-II<br>ANT(3'')-Ia	Carbapenemase (MBL)<br>Aminoglycoside Resistant (AME)	1
17	*Serratia marcescens*	*bla*$_{\text{IMP}}$<br>*bla*$_{\text{OXA-1 like}}$<br>*bla*$_{\text{SEC}}$	AAC(6')-Ic	Carbapenemase (MBL)<br>Aminoglycoside Resistant (AME)	1
18	*Klebsiella pneumoniae*	*bla*$_{\text{TEM-1}}$<br>*bla*$_{\text{SHV}}$ *(multiple alleles including one for an ESBL)*<br>*bla*$_{\text{VIM}}$<br>*bla*$_{\text{MOX}}$	AAC(6')-II<br>AAC(6')-Ip<br>ANT(3'')-Ia<br>APH(2'')-IIa<br>APH(3')-Ia<br>APH(3'')-Ib<br>APH(6)-Id	ESBL + Acquired Case + Carbapenemase (MBL)<br>Aminoglycoside Resistant (AME)	0.5
19	*Klebsiella pneumoniae*	*bla*$_{\text{TEM-1}}$<br>*bla*$_{\text{shv}}$ *(multiple alleles including one for an ESBL)*<br>*bla*$_{\text{KPC}}$<br>*bla*$_{\text{OXA-9 like}}$	AAC(6')-Ib like<br>ANT(3'')-Ia	ESBL + Carbapenemase (KPC)<br>Aminoglycoside Resistant (AME)	0.25
20	*Citrobacter freundii*	*bla*$_{\text{CMY-2 like}}$<br>*bla*$_{\text{NDM}}$<br>*bla*$_{\text{OXA-10 like truncated}}$<br>*bla*$_{\text{CTX-M}}$	AAC(3)-IIa<br>AAC(6')-Ib like truncated<br>ANT(2'')-Ia<br>ANT(3'')-Ia	ESBL + Carbapenemase (MBL)<br>Aminoglycoside Resistant (AME)	0.5
21	*Citrobacter freundii*	*bla*$_{\text{TEM-2}}$<br>*bla*$_{\text{CMY-2 like}}$	AAC(3)-IIa<br>ANT(3'')-Ia	Acquired Pase<br>Aminoglycoside Resistant (AME)	0.25
22	*Klebsiella pneumoniae*	*bla*$_{\text{TEM-1}}$<br>*bla*$_{\text{SHV-1}}$<br>*bla*$_{\text{OXA-48}}$	None	Carbapenemase (OXA-48)<br>Aminoglycoside wild	0.25
23	*Klebsiella pneumoniae*	*bla*$_{\text{CTX-M}}$<br>*bla*$_{\text{SHV-11}}$<br>*bla*$_{\text{NDM}}$<br>*bla*$_{\text{OXA-1 like}}$	AAC(3)-IIa<br>AAC(6')-Ib like truncated<br>AAC(6')-Ib-cr truncated<br>ANT(3'')-Ia	ESBL + Carbapenemase (MBL)<br>Aminoglycoside Resistant (AME)	0.25

Strain	Organism	Genotype beta-lactams (*genes* identified by WGS)	Genotype aminoglycosides (PROTEINS and *genes* identified by WGS)	Beta-lactam and aminoglycoside [GEN, TOB, NET, AMI only])	BMD PLZ MIC (µg/mL)
24	*Klebsiella pneumoniae*	*bla*$_{\text{CTX-M}}$<br>*bla*$_{\text{TEM truncated}}$<br>*bla*$_{\text{SHV truncated}}$<br>*bla*$_{\text{NDM}}$<br>*bla*$_{\text{OXA-1 like}}$<br>*bla*$_{\text{OXA-9 like}}$<br>*bla*$_{\text{DHA truncated}}$	AAC(3)-IIa<br>AAC(6')-Ib like truncated<br>APH(3'')-Ib<br>APH(6)-Id<br><br>*armA*	ESBL + Carbapenemase (MBL)<br>Aminoglycoside Resistant (AME and RNA methylase armA)	>256
25	*Providencia stuartii*	*bla*$_{\text{TEM-1}}$<br>*bla*$_{\text{NDM}}$<br>*bla*$_{\text{VEB}}$<br>*bla*$_{\text{CMY-2 like}}$	AAC(2')-Ia<br>APH(3')-Ia<br>APH(3')-VI<br>APH(3'')-Ib<br>APH(6)-Id ?<br>ANT(3'')-Ia<br><br>*rmtC*	ESBL + Acquired Case +Carbapenemase (MBL)<br>Aminoglycoside Resistant (AME and RNA methylase rmtC)	256
26	*Klebsiella pneumoniae*	*bla*$_{\text{CTX-M}}$<br>*bla*$_{\text{TEM-1}}$<br>*bla*$_{\text{SHV-11}}$<br>*bla*$_{\text{OXA-181}}$<br>*bla*$_{\text{OXA-1 like}}$	AAC(3)-IIa<br>AAC(6')-Ib like truncated<br>AAC(6')-Ib-cr truncated<br>ANT(3'')-Ia<br>APH(3')-Ia<br>APH(3'')-Ib<br>APH(6)-Id<br><br>*rmtF*	ESBL + Carbapenemase (OXA-181)<br>Aminoglycoside resistant (AME and RNA methylase *rmtF*)	>256
27	*Escherichia coli*	*bla*$_{\text{TEM-1}}$<br>*bla*$_{\text{CTX-M}}$	APH(3'')-Ib<br>APH(6)-Id<br>ANT(3'')-Ia<br><br>*rmtB*	ESBL<br>Aminoglycoside resistant (RNA methylase rmtB)	>256
28	*Escherichia coli*	*bla*$_{\text{TEM-1}}$<br>*bla*$_{\text{NDM}}$<br>*bla*$_{\text{VEB}}$<br>*bla*$_{\text{OXA-9 like}}$	AAC(6')-Ib like truncated<br>APH(3'')-Ib<br>APH(6)-Id<br>ANT(2'')-Ia<br>ANT(3'')-Ia<br><br>*armA*	ESBL + Carbapenemase (MBL)<br>Aminoglycoside resistant (AME and RNA methylase armA)	>256
29	*Klebsiella pneumoniae*	*bla*$_{\text{CTX-M}}$<br>*bla*$_{\text{TEM-1}}$<br>*bla*$_{\text{SHV-1}}$<br>*bla*$_{\text{OXA-48}}$<br>*bla*$_{\text{OXA-1 like}}$	AAC(3)-IIa<br>AAC(6')-Ib-cr<br>APH(3')-Ia<br>APH(3'')-Ib<br>APH(6)-Id	ESBL + Carbapenemase (OXA-48)<br>Aminoglycoside Resistant (AME)	0.25
30	*Escherichia coli*	*bla*$_{\text{CTX-M}}$<br>*bla*$_{\text{TEM-1}}$<br>*bla*$_{\text{OXA-48}}$	AAC(3)-IIa<br>ANT(3'')-Ia<br>APH(3'')-Ib<br>APH(6)-Id	ESBL + Carbapenemase (OXA-48)<br>Aminoglycoside Resistant (AME)	0.5
31	*Citrobacter koseri*	*bla* $_{\text{MAL-CKO Like}}$	None	Pase<br>Aminoglycoside wild	0.25
32	*Klebsiella oxytoca*	*bla*$_{\text{OXY}}$	APH(3')-Ia truncated	Beta lactam and Aminoglycoside wild	0.5

Strain	Organism	Genotype beta-lactams (*genes* identified by WGS)	Genotype aminoglycosides (PROTEINS and *genes* identified by WGS)	Beta-lactam and aminoglycoside [GEN, TOB, NET, AMI only])	BMD PLZ MIC (µg/mL)
33	*Klebsiella oxytoca*	*bla*$_{TEM-1}$<br>*bla* $_{OXA-2\ Like\ truncated}$<br>*bla*$_{OXY}$	AAC(6')-IIc<br>ANT(2'')-Ia truncated<br>APH(3')-Ia<br>APH(3'')-Ib<br>APH(6)-Id	Carbapenem Resistant (impermeability)<br>Aminoglycoside Resistant (AME)	1
34	*Klebsiella pneumoniae*	*bla*$_{TEM-1}$<br>*bla*$_{SHV-12}$<br>*bla*$_{OXA-48}$<br>*bla*$_{OXA-1\ like}$	AAC(3)-IIa<br>AAC(6')-Ib-cr<br>ANT(3'')-Ia	ESBL + Carbapenemase (OXA-48)<br>Aminoglycoside Resistant (AME)	0.5
35	*Klebsiella oxytoca*	*bla*$_{TEM-1}$<br>*bla*$_{KPC}$<br>*bla*$_{OXA-9\ like}$<br>*bla*$_{OXY}$	AAC(6')-Ib like<br>ANT(3'')-Ia<br>APH(3')-Ia truncated<br>APH(3'')-Ib<br>APH(6)-Id truncated	Carbapenemase (KPC)<br>Aminoglycoside Resistant (AME)	0.5
36	*Klebsiella oxytoca*	*bla*$_{TEM-1}$<br>*bla*$_{KPC}$<br>*bla*$_{OXA-9\ like}$<br>*bla*$_{OXY}$	AAC(6')-Ib like<br>ANT(3'')-Ia<br>APH(3')-Ia truncated<br>APH(3'')-Ib<br>APH(6)-Id truncated	Carbapenemase (KPC)<br>Aminoglycoside Resistant (AME)	0.25
37	*Klebsiella oxytoca*	*bla*$_{TEM-1}$<br>*bla*$_{KPC}$<br>*bla*$_{OXA-9\ like}$<br>*bla*$_{OXY}$	AAC(6')-Ib like<br>ANT(3'')-Ia<br>APH(3')-Ia truncated<br>APH(3'')-Ib<br>APH(6)-Id truncated	Carbapenemase (KPC)<br>Aminoglycoside Resistant (AME)	0.25
38	*Klebsiella oxytoca*	*bla*$_{OXY}$	None	High level natural Pase (K1)<br>Aminoglycoside wild	0.25
39	*Enterobacter cloacae*	*bla*$_{TEM-1}$<br>*bla*$_{CTX-M}$<br>*bla*$_{OXA-48}$<br>*bla*$_{OXA-1\ like}$<br>*bla*$_{ACT}$	AAC(3)-IIa<br>AAC(6')-Ib-cr<br>ANT(3'')-Ia<br>APH(3'')-Ib truncated<br>APH(6)-Id	ESBL + Carbapenemase (OXA-48)<br>Aminoglycoside Resistant (AME)	0.25
40	*Enterobacter cloacae*	*bla*$_{SHV-12}$<br>*bla*$_{VIM}$<br>*bla*$_{ACT}$	AAC(6')-Ib like<br>ANT(3'')-Ia	ESBL + Carbapenemase (MBL)<br>Aminoglycoside Resistant (AME)	0.25
41	*Escherichia coli*	*bla*$_{TEM-1}$<br>*bla*$_{CTX-M}$<br>*bla*$_{NDM}$<br>*bla*$_{OXA-1\ like}$<br>*bla*$_{OXA-9\ like}$<br>*bla*$_{DHA\ truncated}$	AAC(3)-IIa<br>AAC(6')-Ib like truncated<br>AAC(6')-Ib-cr truncated<br>ANT(3'')-Ia<br><br>*armA*	ESBL + Carbapenemase (MBL)<br>Aminoglycoside resistant (AME and RNA methylase armA)	>256
42	*Klebsiella aerogenes*	*bla*$_{TEM-24}$	AAC(6')-Ib like truncated<br>ANT(3'')-Ia	ESBL<br>Aminoglycoside Resistant (AME)	0.5
43	*Enterobacter cloacae*	*bla*$_{TEM-1}$<br>*bla*$_{SHV-12}$<br>*bla*$_{ACT}$	AAC(6')-Ib like<br>AAC(6')-IIc<br>ANT(3'')-Ia<br>APH(3'')-Ib<br>APH(6)-Id	ESBL<br>Aminoglycoside Resistant (AME)	0.5

Strain	Organism	Genotype beta-lactams (*genes* identified by WGS)	Genotype aminoglycosides (PROTEINS and *genes* identified by WGS)	Beta-lactam and aminoglycoside [GEN, TOB, NET, AMI only])	BMD PLZ MIC (µg/mL)
44	*Enterobacter cloacae*	*bla*$_{CTX-M}$<br>*bla*$_{OXA-1\ like}$<br>*bla*$_{ACT}$	AAC(3)-IIa<br>AAC(6')-Ib-cr<br>ANT(3'')-Ia<br>APH(3'')-Ib<br>APH(6)-Id	ESBL<br>Aminoglycoside Resistant (AME)	0.5
45	*Enterobacter cloacae*	*bla*$_{CTX-M}$<br>*bla*$_{SHV-12}$<br>*bla*$_{ACT}$	AAC(6')-Ib like<br>ANT(2'')-Ia<br>ANT(3'')-Ia	ESBL<br>Aminoglycoside Resistant (AME)	0.25
46	*Enterobacter cloacae*	*bla*$_{ACT}$	None	High Level Case<br>Aminoglycoside wild	0.5
47	*Enterobacter cloacae*	*bla*$_{ACT}$	None	Beta lactam and<br>Aminoglycoside wild	0.5
48	*Providencia stuartii*	*bla*$_{TEM-1}$<br>*bla*$_{SCO}$<br>*bla*$_{ACC}$	AAC(2')-Ia<br>APH(3')-Ia<br>APH(3'')-Ib<br>APH(6)-Id<br>ANT(3'')-Ia	Undetermined for beta-lactams<br>Aminoglycoside wild	1
49	*Citrobacter freundii*	*bla*$_{CMY-2\ like}$	None	High Level Case<br>Aminoglycoside wild	0.5
50	*Klebsiella aerogenes*	None	None	High Level Case<br>Aminoglycoside wild	0.5
51	*Klebsiella pneumoniae*	*bla*$_{TEM-1}$<br>*bla*$_{SHV-11}$<br>*bla*$_{CTX-M}$<br>*bla*$_{OXA-1\ like}$<br>*bla*$_{OXA-48}$	AAC(3)-IIa<br>AAC(6')-Ib-cr	ESBL + Carbapenemase (OXA-48)<br>Aminoglycoside Resistant (AME)	8
52	*Klebsiella pneumoniae*	*bla*$_{TEM-1}$<br>*bla*$_{SHV-11}$<br>*bla*$_{CTX-M}$<br>*bla*$_{OXA-1\ like}$<br>*bla*$_{OXA-48}$	AAC(3)-IIa<br>AAC(6')-Ib-cr	ESBL + Carbapenemase (OXA-48)<br>Aminoglycoside Resistant (AME)	16
53	*Proteus mirabilis*	*bla*$_{TEM-1}$<br>*bla*$_{SHV-12}$	APH(3')-Ia<br>APH(3'')-Ib<br>APH(6)-Id<br>ANT(3'')-Ia	ESBL<br>Aminoglycoside wild	32
54	*Klebsiella aerogenes*	None	None	Undetermined	8
55	*Morganella morganii*	*bla*$_{DHA}$	None	Undetermined	16
56	*Klebsiella pneumoniae*	*bla*$_{TEM-1}$<br>*bla*$_{SHV-11}$<br>*bla*$_{KPC}$<br>*bla*$_{OXA-9\ like}$	AAC(6')-Ib like<br>APH(3'')-Ib<br>APH(6)-Id<br>ANT(3'')-Ia	Carbapenemase (KPC)<br>Aminoglycoside Resistant (AME)	2
57	*Klebsiella aerogenes*	*bla*$_{SHV-12}$	AAC(6')-Ib like<br>AAC(6')-IIc<br>ANT(3'')-Ia	ESBL + Carbapenem Resistant (impermeability)<br>Aminoglycoside Resistant (AME)	1

Strain	Organism	Genotype beta-lactams (*genes* identified by WGS)	Genotype aminoglycosides (PROTEINS and *genes* identified by WGS)	Beta-lactam and aminoglycoside [GEN, TOB, NET, AMI only])	BMD PLZ MIC (µg/mL)
58	*Citrobacter freundii*	*bla*$_{CMY-2\ like}$	None	Beta lactam and Aminoglycoside wild	0.5
59	*Morganella morganii*	*bla*$_{DHA}$	None	High Level Case Aminoglycoside wild	8
60	*Morganella morganii*	*bla*$_{DHA}$	None	Beta lactam and Aminoglycoside wild	2
61	*Proteus mirabilis*	*bla*$_{TEM-1}$<br>*bla*$_{CMY-2\ like}$<br>*bla*$_{OXA-2\ like}$	AAC(3)-IIa<br>AAC(6')-Ib like<br>ANT(3'')-Ia	ESBL + Acquired Case Aminoglycoside Resistant (AME)	8
62	*Proteus vulgaris group*	None	None	Acquired Pase Aminoglycoside wild	4
63	*Serratia marcescens*	None	AAC(6')-Ic	Undetermined for beta-lactams Aminoglycoside Resistant (AME)	1
64	*Klebsiella pneumoniae*	*bla*$_{SHV}$ *(multiple alleles including one for an ESBL)*<br>*bla*$_{OXA-10\ like}$	APH(3'')-Ib<br>APH(6)-Id truncated<br>ANT(3'')-Ia truncated	ESBL + Carbapenem Resistant (impermeability) Aminoglycoside undetermined	8
65	*Serratia marcescens*	*bla*$_{SEC}$	None	Beta lactam and Aminoglycoside wild	1
66	*Proteus vulgaris group*	None	None	Acquired Pase or wild (cefuroximase) Aminoglycoside wild	4
67	*Proteus vulgaris group*	None	None	Acquired Pase Aminoglycoside wild	2
68	*Klebsiella pneumoniae*	*bla*$_{TEM-1}$<br>*bla*$_{SHV}$ *(multiple alleles including one for an ESBL)*<br>*bla*$_{VEB}$<br>*bla*$_{OXA-10\ like}$	AAC(6')-Ib like<br>APH(3')-Ia<br>APH(3'')-Ib<br>APH(6)-Id<br>ANT(2'')-Ia<br>ANT(3'')-Ia<br><br>*rmtB*	ESBL<br>Aminoglycoside Resistant (AME and RNA methylase rmtB)	>256
69	*Escherichia coli*	*bla*$_{TEM-1}$	APH(3'')-Ib<br>APH(6)-Id	Acquired Pase Aminoglycoside undetermined	4
70	*Proteus vulgaris group*	None	None	Undetermined	2
71	*Klebsiella pneumoniae*	*bla*$_{SHV-11}$	None	Acquired Pase Aminoglycoside undetermined	4
72	*Proteus mirabilis*	*bla*$_{TEM-1}$	APH(3')-Ia<br>APH(3'')-Ib<br>APH(6)-Id<br>ANT(3'')-Ia	Acquired Pase Aminoglycoside wild	4

Strain	Organism	Genotype beta-lactams (*genes* identified by WGS)	Genotype aminoglycosides (PROTEINS and *genes* identified by WGS)	Beta-lactam and aminoglycoside [GEN, TOB, NET, AMI only])	BMD PLZ MIC (µg/mL)
73	*Klebsiella oxytoca*	$bla_{\text{TEM-1}}$<br>bla_{OXY}	ANT(3'')-Ia	High level Natural Pase (K1)<br>Aminoglycoside undetermined	2
74	*Proteus vulgaris group*	None	None	High level cefuroximase<br>Aminoglycoside wild	4
75	*Escherichia coli*	$bla_{\text{TEM-1}}$	None	Acquired Pase<br>Aminoglycoside undetermined	4
76	*Proteus mirabilis*	$bla_{\text{TEM-1}}$<br>$bla_{\text{CARB-1 like}}$	AAC(3)-Id<br>APH(3')-Ia<br>APH(3'')-Ib<br>APH(6)-Id<br>ANT(3'')-Ia	Inhibitor resistant Pase<br>Aminoglycoside Resistant (AME)	2
77	*Klebsiella pneumoniae*	$bla_{\text{SHV-1}}$<br>bla_{KPC}	*armA*	Carbapenemase (KPC)<br>Aminoglycoside Resistant (RNA methylase armA)	>256
78	*Escherichia coli*	$bla_{\text{TEM-1}}$<br>$bla_{\text{CTX-M}}$<br>$bla_{\text{OXA-1 like}}$	AAC(3)-IIa<br>AAC(6')-Ib-cr<br>ANT(3'')-Ia<br>*rmtB*	ESBL<br>Aminoglycoside Resistant (AME and RNA methylase rmtB)	>256
79	*Escherichia coli*	$bla_{\text{TEM-1}}$<br>$bla_{\text{CTX-M}}$	AAC(3)-IIa<br>APH(3')-IIa<br>ANT(3'')-Ia<br>*rmtB*	ESBL<br>Aminoglycoside Resistant (AME and RNA methylase rmtB)	>256
80	*Escherichia coli*	$bla_{\text{TEM-1}}$<br>$bla_{\text{CTX-M}}$	AAC(3)-IIa	ESBL<br>Aminoglycoside Resistant (AME)	1

***AME = Aminoglycoside Modifying Enzymes**

Pase = penicillinase

Case = cephalosporinase
